# Supplementary material for: Single domain antibodies against enteric pathogen virulence factors are active as curli fiber fusions on probiotic E. coli Nissle 1917
Source: PLoS Pathog. 2022 Sep 15;18(9):e1010713. doi: 10.1371/journal.ppat.1010713 (PMC9477280; doi:10.1371/journal.ppat.1010713)
Supplement: S1 Table — (DOCX) [file ppat.1010713.s001.docx]

**S1 Table: Strains and plasmids**

| 1. **Strains** | | |
| --- | --- | --- |
| **Strain** | **Description/comments** | **Reference** |
| Mach1 | *E. coli*, str.K-12 F^-^ ϕ80(*lac*Z) ΔM15 Δ*lac*X74 *hsd*R(r_K_^-^m_K_^+^) Δ*recA*1398 *end*A1 *ton*A, cloning strain | Thermo Fisher Scientific |
| MC1061 | *E. coli*, str. K-12 F^–^ λ^–^ Δ(ara-leu)7697 [araD139]B/r Δ(codB-lacI)3 galK16 galE15 e14^–^ mcrA0 relA1 rpsL150(Str^R^) spoT1 mcrB1 hsdR2(r^–^m^+^) | Casadaban *et al.*,  1980 [1] |
| PBP8 | *E. coli* Nissle 1917, Δ*csgBACDEFG*::*Cm^R^* | Praveschotinunt *et al.*, 2018 [2] |
| 2457T | *Shigella flexneri* 2a strain 2457T | Mills *et al.*,  1992 [3] |
| E2348/69 | *E. coli* strain O127:H6/**EPEC** ; widely used as a model for EPEC infection | Iguchi *et al*.,  2009 [4] |
| EDL933 | *E. coli* strain O157:H7/**EHEC**, *stx1 stx2 eae espP/pssA hly_EHEC_*) | O’Brien *et al.*, 1983 [5] |
| E22 | *E. coli* strain O103:K-H2/**REPEC**, *rha*- | Camguilhem *et al*.,  1989 [6] |
| 3014-2 | *E. coli* strain O153:H-/**REHEC**, sorbitol+ | García *et al.*,  2002 [7] |
| DBS770 | *Citrobacter rodentium,* derivative of strain ICC168, chloramphenicol resistant. Lysogenized with Stx2dact-producing phage ϕ1720. Produces Shiga toxin. | Mallick *et al*.,  2012 [8] |
| DBS771 | *Citrobacter rodentium*, chloramphenical resistant, kanamycin resistant, *stx2dact*. DBS770 with KanR cassette inserted into prophage *stx* genes. Does not produce Shiga toxin. | Mallick *et al*.,  2012 [8] |
| E10 | *E. coli* strain O119:H6/EPEC wt | Girón *et al*., 2002 [9] |

| 1. **Plasmids** | | |
| --- | --- | --- |
| **Plasmid** | **Description/comments** | **Reference** |
| pL6FO | CsgA-VHH expression vector, contains synthetic curli operon *csgBACEFG* under an IPTG-inducible promoter. | Kan *et al.*, 2019 [10] |
| pGex-2t | Vector for EHEC intimin GST-Int465 (*eaeA*, EDL933 (C-terminal 465aa, bp1636-3082) (Yu and Kaper, 1992 [11])); EPEC GST-TirM (E2348/69 TirM 120aa) (Burland *et al.*, 1998 [12]); REPEC GST-TirM (E22 TirM 120aa) | Pharmacia, [Liu *et al.*, 1999](https://doi.org/10.1046/j.1365-2958.1999.01574.x) [13] |
| pDEST15 | Vector for *C. rodentium* intimin GST-Int400C (*eaeA*, *Citrobacter rodentium* strain DBS100 (C-terminal 400aa)) | Thermo Fisher Scientific |
| pGex-4t2 | Vector for EPEC intimin (*eaeA*, E2348/69 (C-terminal 400aa)); REPEC intimin (*eaeA*, E22 (C-terminal 400aa)) | Pharmacia |
| pET15b | Vector for EPEC His-TirM (E2348/69 TirM 120aa) (Burland *et al.*, 1998 [12]) | Novagen |
| pDest17 | Vector for REPEC His-TirM (E22 TirM 120aa); *C. rodentium* His-TirM (DBS100 TirM 120aa) | Thermo Fisher Scientific |
| pMalc2 | Vector for EHEC intimin MBP-Int395 (*eaeA*, EDL933 (C-terminal 395aa, bp1618-2082) (Liu *et al.*, 2002 [14])) | New England Biolabs, [Liu *et al.*, 1999](https://doi.org/10.1046/j.1365-2958.1999.01574.x) [13] |
| pUC19 | Vector for intimin expression in MC1061 (*eaeA*, full-length, EDL933 bp -30-3011; *eaeA*, full-length, JPN15 bp 1614-4537) (Yu and Kaper, 1992 [11]) | Yanisch-Perron *et al.*, 1985 [15] |
| pET23 | Vector for expression of recombinant VHHs | Vrentas *et al.*, 2010 [16] |

References

1. Casadaban MJ, Cohen SN. Analysis of gene control signals by DNA fusion and cloning in Escherichia coli. Journal of Molecular Biology. 1980;138(2):179-207.

2. Praveschotinunt P, Dorval Courchesne N-M, den Hartog I, Lu C, Kim JJ, Nguyen PQ, et al. Tracking of Engineered Bacteria In Vivo Using Nonstandard Amino Acid Incorporation. ACS Synthetic Biology. 2018;7(6):1640-50.

3. Mills JA. Molecular characterization of a noninvasive opaque colonial variant of Shigella flexneri. 1992.

4. Iguchi A, Thomson NR, Ogura Y, Saunders D, Ooka T, Henderson IR, et al. Complete Genome Sequence and Comparative Genome Analysis of Enteropathogenic Escherichia coli O127:H6 Strain E2348/69. Journal of Bacteriology. 2009;191(1):347.

5. Brien AD, LaVeck GD. Purification and characterization of a Shigella dysenteriae 1-like toxin produced by Escherichia coli. Infection and immunity. 1983;40(2):675.

6. Camguilhem R, Milon A. Biotypes and O serogroups of Escherichia coli involved in intestinal infections of weaned rabbits: clues to diagnosis of pathogenic strains. Journal of Clinical Microbiology. 1989;27(4):743.

7. García A, Marini RP, Feng Y, Vitsky A, Knox KA, Taylor NS, et al. A Naturally Occurring Rabbit Model of Enterohemorrhagic Escherichia coli–Induced Disease. The Journal of infectious diseases. 2002;186(11):1682-6.

8. Mallick EM, McBee ME, Vanguri VK, Melton-Celsa AR, Schlieper K, Karalius BJ, et al. A novel murine infection model for Shiga toxin–producing Escherichia coli. The Journal of Clinical Investigation. 2012;122(11):4012-24.

9. Girón JA, Torres AG, Freer E, Kaper JB. The flagella of enteropathogenic Escherichia coli mediate adherence to epithelial cells. Molecular microbiology. 2002;44(2):361-79.

10. Kan A, Birnbaum DP, Praveschotinunt P, Joshi NS. Congo Red Fluorescence for Rapid In Situ Characterization of Synthetic Curli Systems. Applied and Environmental Microbiology. 2019;85(13):e00434-19.

11. Yu J, Kaper JB. Cloning and characterization of the eae gene of enterohaemorrhagic Escherichia coli O157:H7. Molecular microbiology. 1992;6(3):411-7.

12. Burland V, Shao Y, Perna NT, Plunkett G, Blattner FR, Sofia HJ. The complete DNA sequence and analysis of the large virulence plasmid of Escherichia coli O157:H7. Nucleic Acids Research. 1998;26(18):4196-204.

13. Liu H, Magoun L, Luperchio S, Schauer DB, Leong JM. The Tir-binding region of enterohaemorrhagic Escherichia coli intimin is sufficient to trigger actin condensation after bacterial-induced host cell signalling. Molecular microbiology. 1999;34(1):67-81.

14. Liu H, Radhakrishnan P, Magoun L, Prabu M, Campellone KG, Savage P, et al. Point mutants of EHEC intimin that diminish Tir recognition and actin pedestal formation highlight a putative Tir binding pocket. Molecular microbiology. 2002;45(6):1557-73.

15. Yanisch-Perron C, Vieira J, Messing J. Improved M13 phage cloning vectors and host strains: nucleotide sequences of the M13mp18 and pUC19 vectors. Gene. 1985;33(1):103-19.

16. Vrentas CE, Moayeri M, Keefer AB, Greaney AJ, Tremblay J, O'Mard D, et al. A Diverse Set of Single-domain Antibodies (VHHs) against the Anthrax Toxin Lethal and Edema Factors Provides a Basis for Construction of a Bispecific Agent That Protects against Anthrax Infection. Journal of Biological Chemistry. 2016;291(41):21596-606.
