# Supplementary material for: Single domain antibodies against enteric pathogen virulence factors are active as curli fiber fusions on probiotic E. coli Nissle 1917
Source: PLoS Pathog. 2022 Sep 15;18(9):e1010713. doi: 10.1371/journal.ppat.1010713 (PMC9477280; doi:10.1371/journal.ppat.1010713)
Supplement: S2 Table — Complementarity determining regions (CDR1, 2 and 3) are highlighted and appear in order from left to right. (PDF) [file ppat.1010713.s002.pdf]

S2 Table: Aligned sequences of VHHs binding pathogenic *E. coli* virulence factors. Complementarity determining regions (CDR1, 2 and 3) are highlighted and appear in order from left to right.

Anti-Fla:

JUV-B11: SGGGLAQPGGSLRLSCTSTGHT-LDDYAIGWFRQAPGKERERVACASASGI-TTNYADSVKGRFTISRDKAKNMVYLQMNSLQPEDTAVYYCAA---TPYYGDVCVRAAFESRGQGTQLTVSS

JUV-C4: TGGGLVQAGGSLTLSCVASGRA-VSSFAMGWFRQIPGREQRDFVAFIGDYGLTTYYANSVKGRFTISRSNAENTLYLQMNSLEFEDAAVYFCAA--RDAYRSGTTNPSAYDYWGQGTQVTVSS

JUV-E8: SGGGLVQAGGSLRLSCAASGRT-SSTYTMGWFRQAPGKEREFAAAIRSSGS-GTYYADSVKGRFTISRDGAKNTVYLQMNSLKPEDTAVYYCAA--RGNPIYSVYDVRTYDLWGQGTQVTVSS

JUV-G8: TGGGLVQPGGSLRLSCAASGSI-VSFNAMVWYREAPGKQREWVAQITPSSK--TMYKDSVKGRFTISSDNAKNMVYLQMNNLKPEDTAVYYCNGD-------------RGVAWGPGTQVIVSS

JUV-H5: SGGGLVQPGGSLRLSCAASEMS-FSIKRMGWFREAPGKPREWVAQITPAGS--TNYAETVKGRFTISRDNAKNTVYLQMNSLKPDDTAVYYCNT-------------LPGIAWGQGTQVIVSL

JWU-F3: SGGGLVQPGGSLRLTCVSSLSD-FRLTNMAWYRQTPGSERDVVAGISPNGI--TSYHASVQDRFNISRDNARKTLFLQMNSLKPEDSGVYYCNI-----------RWGSLLPWGQGTQVTVSP

JWU-H4: TGGGSVQPGGSLRLSCAPSGFS-LAYYGVGWFRQAPGKEREALACISRFGD-DTYYADSAKGRFTVSRDNAKNTVYLEMNNLKPEDTGVYYCAAGWVVVTDESCGSGDAYNYWGRGTQVTVSS

JXE-B1: TGGGLVQAGDSLRLSCAASSGRNFSNYATGWFRQAPGKEQEFVASISRSGR-STYYADSAKGRFTISRDNARNTVYLQMNSLKPEDTADYYCAAHETQWPNGLGWVRGFYDLWGQGTQVTVSS

Anti-intimin:

JWS-H4: SGGGLVQAGGSLRLSCTTSASIFSGYRMGWFRQAPGKQREFVASIADG-QNTPYADSVMGRFTISRDNAKNTVYLQMSNLKPEDTAIYYCKS-----------------WGYTDWGQGTQVTVSS

JWT-C1: S-GGLVQPGGSLRLSCAASGFTLANSAIGWFRQAPGKGREAVSCISTSAGTTNYASSVKGRFTTARDNAKNMAYLQMDNLKSGDTGVYECAA------GWSIDCSGYILPAADVWGQGTQVTVSS

JWU-D8: TGGGLVQPGGSLRLSCAASGFYFSGYWMHWVRQVQGQGLKWVSGINIDDTKSSYTDSVKGRFTISRDNTKNTLYLQMDSLQPEDTGVYYCAR-------DRRAGQISGGYDPDYRGQGTQVTVSS

JWU-G8: SGGGLVQPGGSLRLSCATSGFYFAGYWMHWVRQVPGQGLEWVSGIDLGSTMLNYRDSVKGRFSISRDNAKNTVYLQMHSLKPEDTALYFCAR-------DRRAGATSGGYDPDVRGQGTQVTVSS

JXN-E2: T-GGLVPPGGSLQLSCTSSGFFLDYLGVAWFRQAPGNEREGVSCIDYTGDNIAYASSMKGRATISRDKDANTVTLEMNGLKPEDTAVYYCAAHRSATTYADGKYRCPLENEYDYWGQGIQVTVSS

Anti-Tir:

JVB-C6: TGGGLVQPGGSLTLSCAASGFSITENAMGWARQVPGKGLEWVSLV---------YSGGNTYYAESIEGRFTISRDNAKNTVYLRMTSLKPEDTGVYYCAA----REAVRVAGPPADVWGQGTQVTVSS

JVB-G4: SGGGLVEAGGSLRLSCSASGRASGDGHLGWFGDGALAWFRQAPGKEREYVAAVGHSGTDTYYSDSVKGRFTISRDNAKSMGYLQMDNLRPDDTGIYYCAL------DLHLGQPGYDYWGQGTQVIVSS

JVB-G8: TGGGLVQAGGSLRLSCVASGFTFNDYVLTWFRQAPGKGREGVASI--------SPAFGNTFYADSVKGRFTITSDSAKKQVCLQMNNLKSEDTAVYFCAADPTVNFGPVLRAENYRHWGQGTQVTVSS

JVC-C6: SGGGLVQAGGSLRLSCAASGVTFVDYATGWFRQAPGKEREGIACI--------SNGASGSFVVNSVKGRFTTSTDNAKNTAYLQMNDLKPEDTATYYCAL-LGRTGGHCTDPNEYSYWGQGTQVTVSS

JVC-D10: T-GGLVQTGDSLTLSCVVSGRGFCDWAMAWFRQAPGKEREKVSAI--------GWSGQDTYYSEPAKGRFTISRDNAKNTVWLRMTNLKSEDTAVYYCAA-------ATRAYADYDYWGQGTQVTVSS

JVC-E5: TGGGTVQAGSSLRLSCTASGFTFTDAAMGWFRQAPGKEREFVAAI--------NWDSANKYYADSVKGRFTISRDNAKNTVYLEMTALKPEDTADYICAG--DAKLGHVATSDVWRFWGQGTQVTVSS

JVA-A1: S-GGSVEAGDSLRLSCAASGRGFGDGALAWFRQAPGKEREYVAAV--------GHSGTDTYYADSVKGRFTISRDNAKNMGYLQMDSLRPDDTGVYYCAL------DLHLGQPGYDYWGQGTQVTVSS

JVA-C8: SGGGLVQAGGSLRLSCAASGFTFVDYGIAWVRQAPGKEREGVSCV--------STSNRSQWYADSVKGRFTISSDNAKNTVYLQMDNLKPEDTAVYYCTR----IDVGDNCRDGGGYRGQGTQVTISS

JVA-C9: TGGGLVQAGGSLRLSCVDSGRTFGDNAMGWFRQAPGKEREYVAAI--------GYNGDSTYYLNSVKGRFTISRDNAKNTLYLQMNSLQLEDTGVYICAA------KTRVTTKEYDFWGQGTQVTVSS

JVA-D4: TGGGLAQAGGSLRLSCATSGFTFADYAIGWFRQAPGKEREGVACI--------STSDNGQYSADSVKGRFTISKDNAKKTVYLQMNSLKPDDTAIYYCHL--IDVGSNCRKGKDDGFWGQGTQVTVSS

JVA-F6: SGGGLVQAGDSLRLSCVTSGRSFSEAAMGWFRQAPGKERELMTSI--------GWNGDRTYYADSVKGRFTISRDNAKNTVYLQMNGLTPNDTAVYYCAA-------AVRASKGYEYWGQGTQVTVSS

JVA-D11: SGGGLVQAGGSLRLSCAASGFTFNDYAKAWFRRAPGKEREGISAI--------SSMGESTFYADSVKGRFTISSDSAKNTVYLQMNSLKPEDTAVYYCAADPTVKWGHVLREENYDYWGQGTQVTVVS

JVA-E10: SGGGLVQAGGSLRLSCVASGRTFSDYAMGWFRQAPGKEREFVAAI--------SWNGGSTYYADSVKGRFTISRDNAKNTMYLQMNSLKSEDTAVYYCIS-EGRGLRQVKTATDWEYWGQGTQVTVSP

JVA-G1: SGGGLAQAGGSLKLSCVASGFTFADYAIGWFRQAPGKEREGVACI--------SNSVGSTYYSDSVKGRVTISSDNVKKTVYLQMNSLKPEDTAFYYCAL--IDHGMNCRNSNGIHYWGKGTLVTVSS

Anti-EspA:

JXF-D7: SGGGLVQTGGSLRLSCSASGFALEYYAVGWFRQAPGKEREGVSCF--SGSDGSKFHAQFVKGRFTISLDKEKNTVDLTMNNLKPEDTAVYYCAVA-GPSDHQCDLGMTWYHRWGQGTQVTVSS

JYB-B1: TGGGLVQAGGSLRLSCAISGFSLGDYAIGWFRQAPEKEREGVAFS--GSLGNTY-YPDSMKGRFTISRDDAENAVYLEMKNLKPEDTAVYRCTS----GGTFDAVVLGLSTYWGQGTQVTVSS

JYB-B8: TGGGLVQPGGSLRLSCAASGFTFSTYWMYWVRQAPGKGLEWVSTI--DTGGSDTYYADSVKGRFTISRDNVKNTLYLQMDNLKPEDTALYYCGS---SRDIVIVTTLRDFDYWGQGTQVTVFS

JYB-D1: TGGGLVQAGGSLRLSCAASGNTFSYTAMAWFRQAPGKQRELVARI--SSGRGPTKYADSVKGRFTISRDNTLNTVWLQMNDLKPEDTAVYFCNT----LKYSGESSYIAGDSWGQGTQVTVSS

JYB-H4: SGGGLVQPGGSLRLSCAASGVTLEYYAIGWFRQVPGKEREGVSCI--STSGAGTNYADSVKGRFTISKDNAKNTVYLQMNSLKPEDTAVYYCAA-RDFTEVPIGGCEWEYDYWGQGTQVTVSS

JYB-H6: TGGGLVQPGGSLRLSCVASGFILDAYTIGWFRQAPGKEREGVASI--NGSGFSTNYADSVKGRFTISRDNAKNTVWLQMNSLKPEDTAVYYCAA--ALGLLTPRLESTPFDDWGQGTQVTVSS

JYF-D8: SGGALVQPGGSLRLSCAASGINLDYYAIGWFRQAPGKERKGVSCI-SHVDDDRIYYSDSVKGRFTISRDNAKNTVYLQMNSLEPEDTAVYYCATA-GPSDYPCDLELWTYRRWGQGTQVTVSS

JXF-H9: SGGGLAQTGGSLRLSCAASGFRLEYYAVGWFRQAPGKEREGVSCL--SGSDGSTYNAEFAKGRFTISRDDAKNTVYLLMNSLKPEDTAVYYCAVA-GPSDYQCDLGTRWYHRWGQGTVVTVSA

JXF-C4: SGGGLVQAGGSLRLSCVAYGRTANTFKEYAMGWFRHNPGEDHEFVGGISQNGDEAYFDDSVKGRFTPSRDNAKNTMYLQMNSLRPEDTAAYYCVAQRSSERLVGDMYSAMDSWGKGTLVTVST
