# Supplementary figures and images for: Single domain antibodies against enteric pathogen virulence factors are active as curli fiber fusions on probiotic E. coli Nissle 1917
Source: PLoS Pathog. 2022 Sep 15;18(9):e1010713. doi: 10.1371/journal.ppat.1010713 (PMC9477280; doi:10.1371/journal.ppat.1010713)

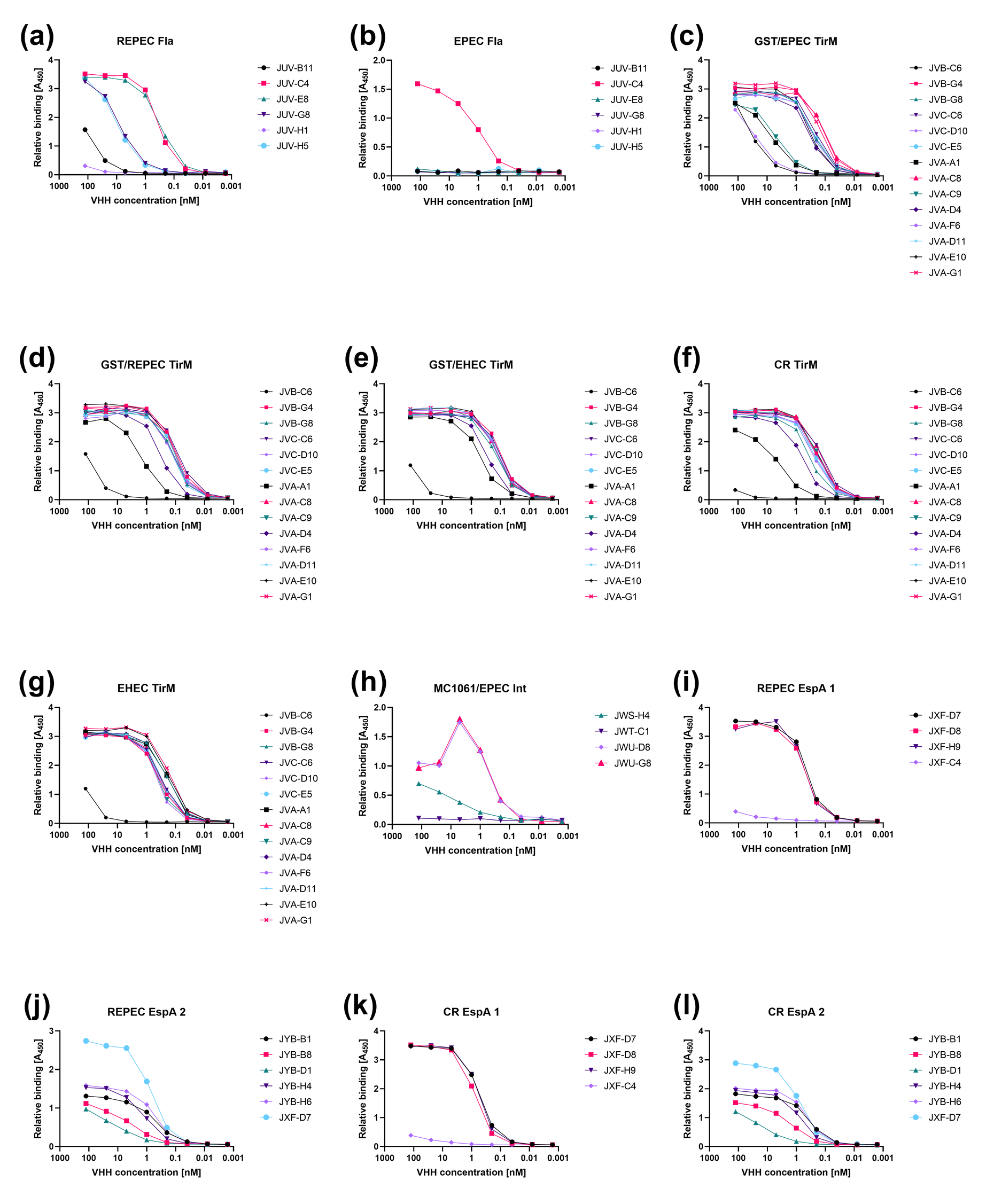

Supplement: S1 Fig — Antigens used were homologues of Fla (a-b), Tir (c-g), Int (h) or EspA (i-l) corresponding to either EPEC (b, c, h), EHEC (e, g), REPEC (a, d, i, j) or C. rodentium (f, k, l). In each assay, antigen was either directly added to the plate in purified form (a-b, e-g, i-l), bound to the plate by an adsorbed noncompeting VHH (c-d), or displayed on the surface of MC1061 (h). (TIFF) [file ppat.1010713.s003.tiff]

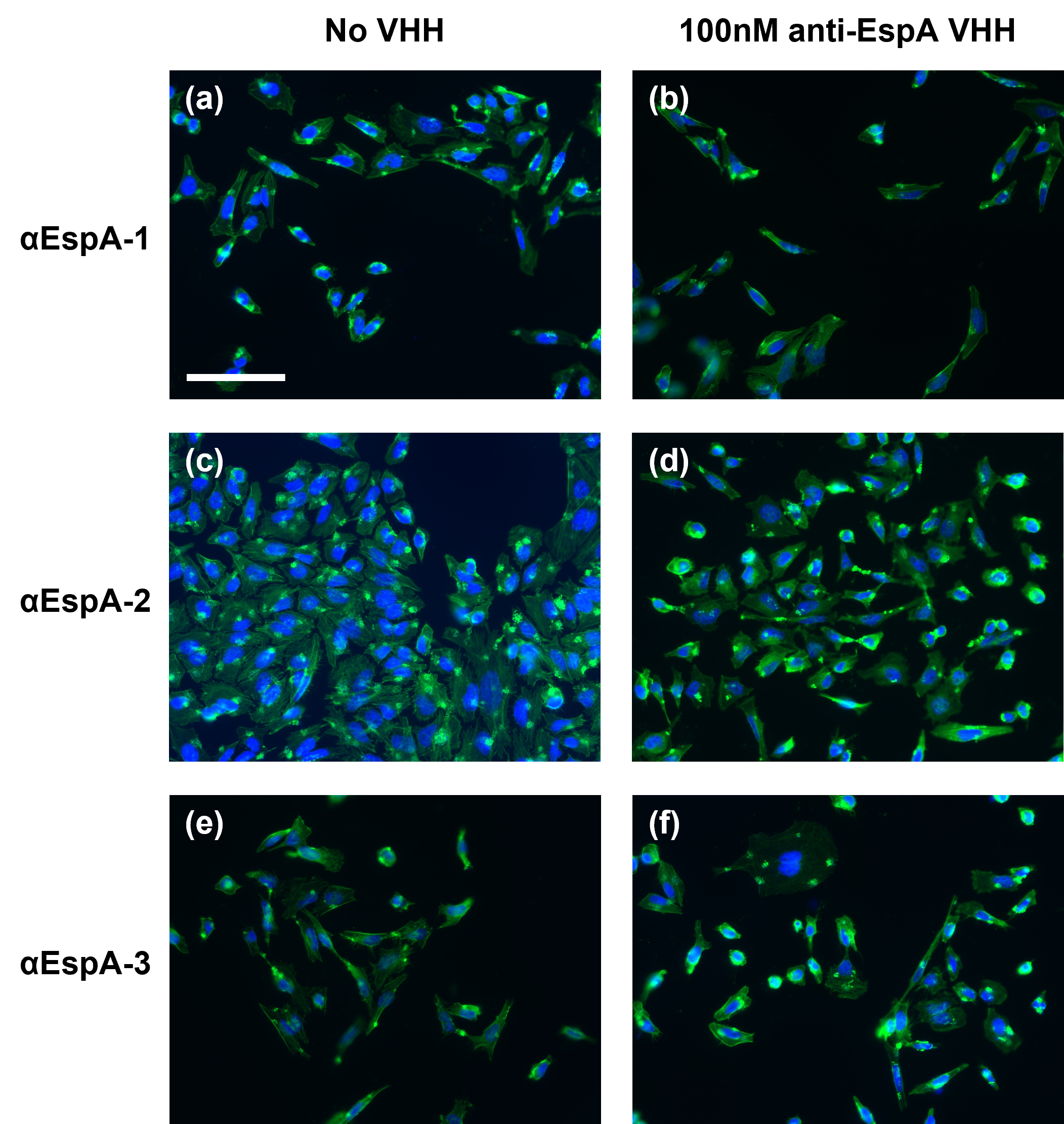

Supplement: S2 Fig — HeLa cells were exposed to EPEC incubated with VHH, fixed and stained with DAPI (blue) and Alexa Fluor-488 Phalloidin (green). Similar to the “no VHH” negative control (a, c, e), all anti-EspA VHHs tested (b, d, f) resulted in the formation of pedestals (though not all anti-EspA VHHs were tested) (scale bar = 100 μm). (TIFF) [file ppat.1010713.s004.tiff]

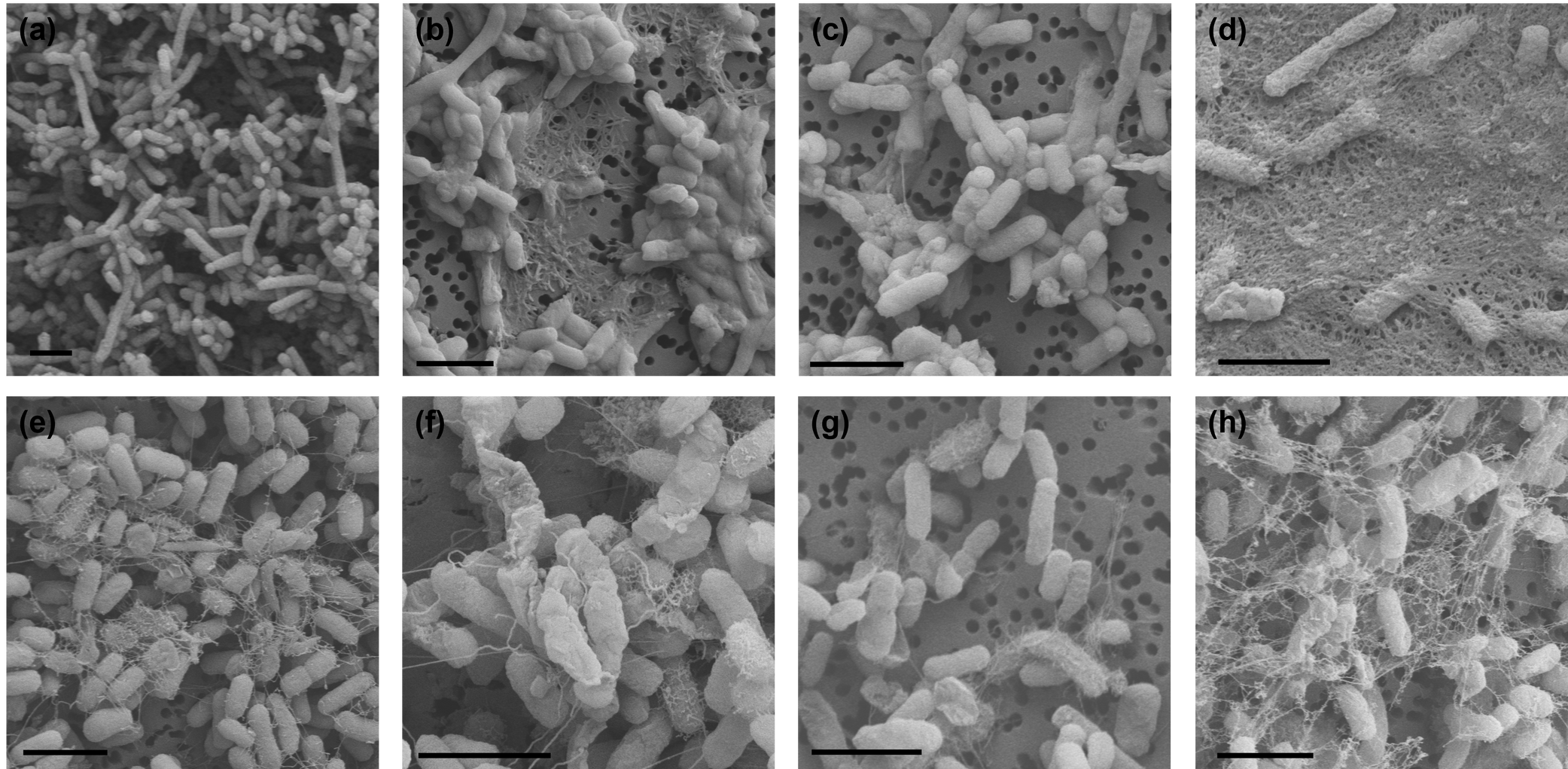

Supplement: S3 Fig — (a) PBP8 with no plasmid, expressing no curli fibers. (b-h) PBP8 expressing CsgA-VHH, exhibiting a range of fiber morphologies. (b) CsgA-αStx2, (c) CsgA-αInt-12, (d) CsgA-αInt-17, (e) CsgA-αFla-3, (f) CsgA-αFla-4, (g) CsgA-αIpaD-1, (h) CsgA-αgp900-2 (scale bar = 2 μm). (TIFF) [file ppat.1010713.s005.tiff]

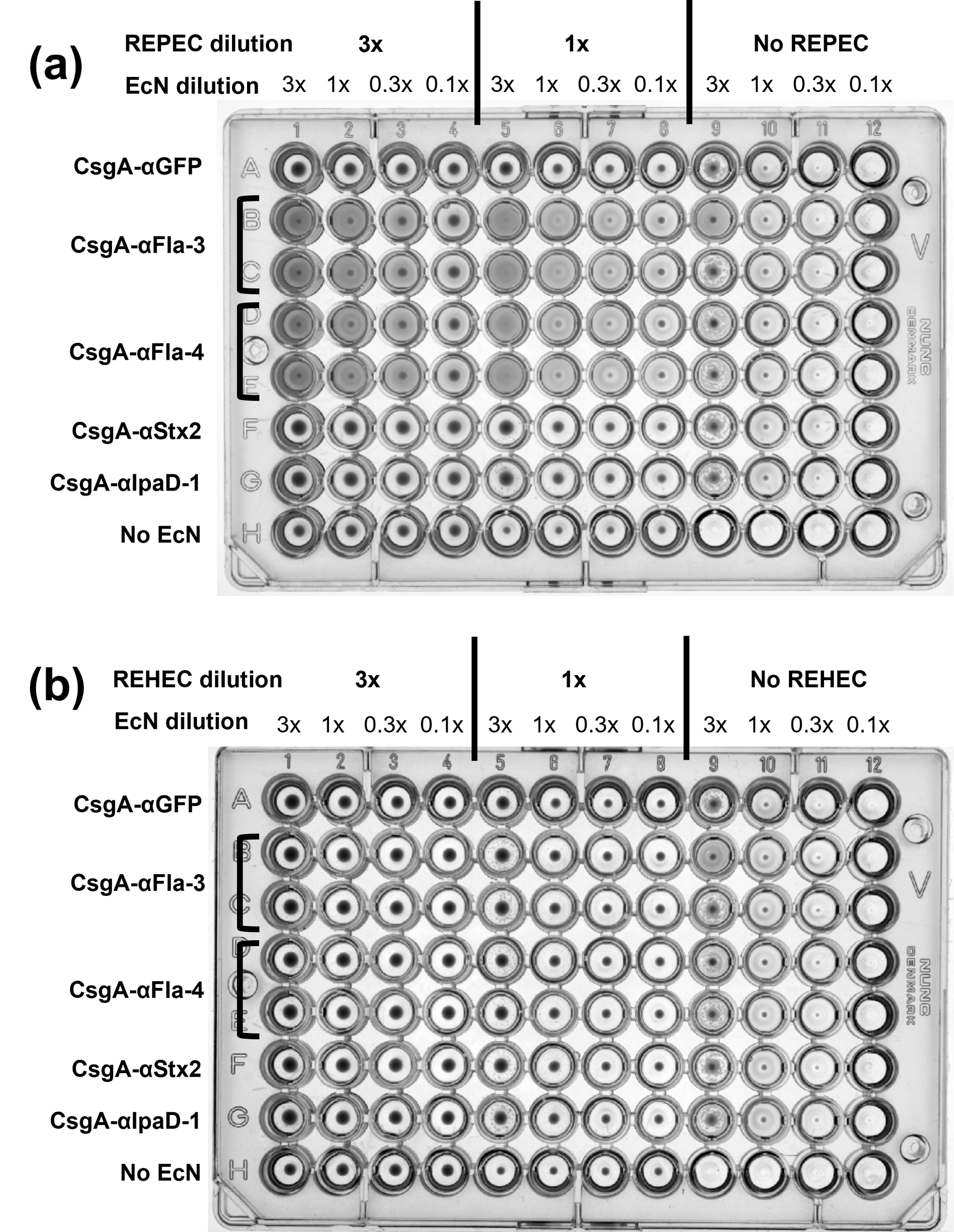

Supplement: S4 Fig — Suspensions of PBP8 expressing CsgA-VHH were mixed with either REPEC (a) or REHEC (b) and allowed to settle overnight in conical 96-well plates. Aggregation was only observed when REPEC was mixed with CsgA-αFla-3 and -4. (TIFF) [file ppat.1010713.s006.tiff]

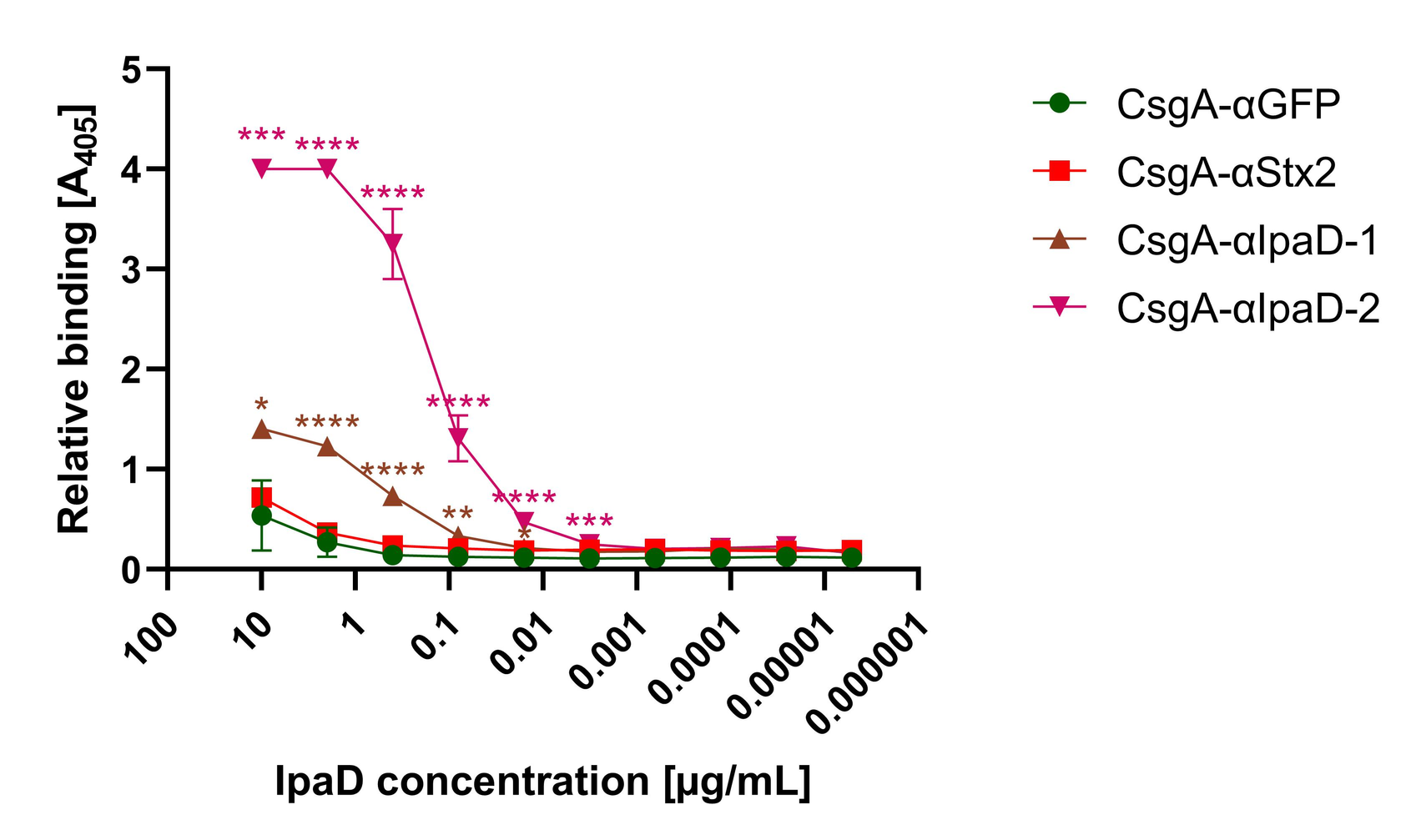

Supplement: S5 Fig — ELISA demonstrated the ability of CsgA-αIpaD to bind soluble IpaD. PBP8 was adsorbed onto a well plate, followed by incubation with varying IpaD concentrations. Binding of IpaD to the adsorbed PBP8 was then detected by a specific non-competing VHH (JMK-H2, Barta et, al., 2017 [20]), followed by an anti-Etag IgG-HRP conjugate. PBP8 expressing either CsgA-αIpaD-1 or CsgA-αIpaD-2 significantly outperformed the off-target negative control (CsgA-αGFP). Data presented as mean ± SD. Two-way ANOVA (P < 0.0001) was performed to test the presence of difference between conditions, P-values calculated by Welch’s t-test. * P < 0.05; ** P < 0.01; *** P < 0.001; **** P < 0.0001. (TIFF) [file ppat.1010713.s007.tiff]

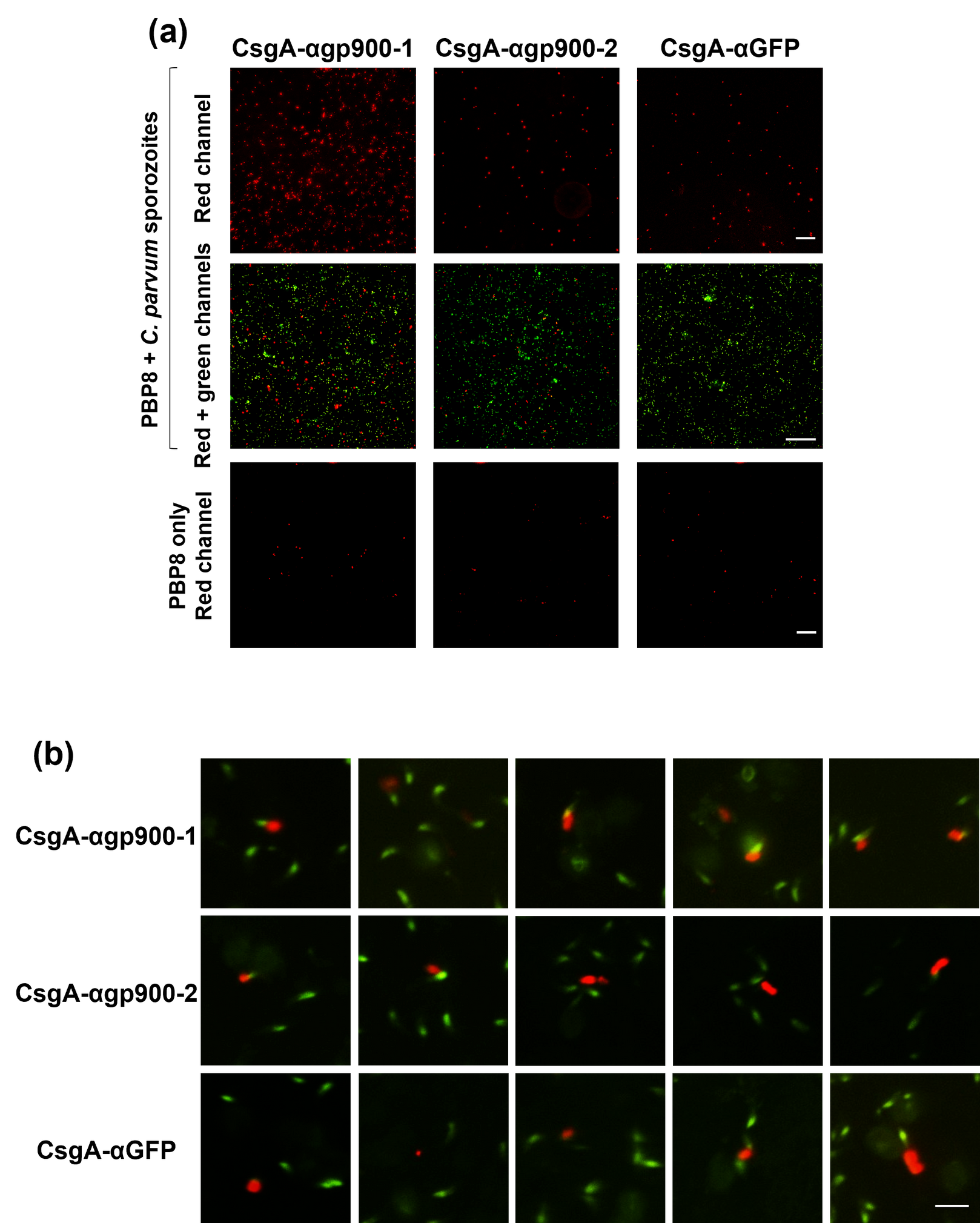

Supplement: S6 Fig — (a) Fluorescent micrographs demonstrating increased attachment of PBP8 (red) to C. parvum sporozoites, counterstained in green in the bottom panels (scale bar = 50 μm). (b) While PBP8 (red) expressing CsgA-ɑgp900-1 (and to a lesser extent CsgA-ɑgp900-2) consistently colocalize with sporozoites (green), the CsgA-ɑGFP negative control was often observed away from the green fluorescent foci, consistent with nonspecific binding (scale bar = 5 μm). (TIFF) [file ppat.1010713.s008.tiff]

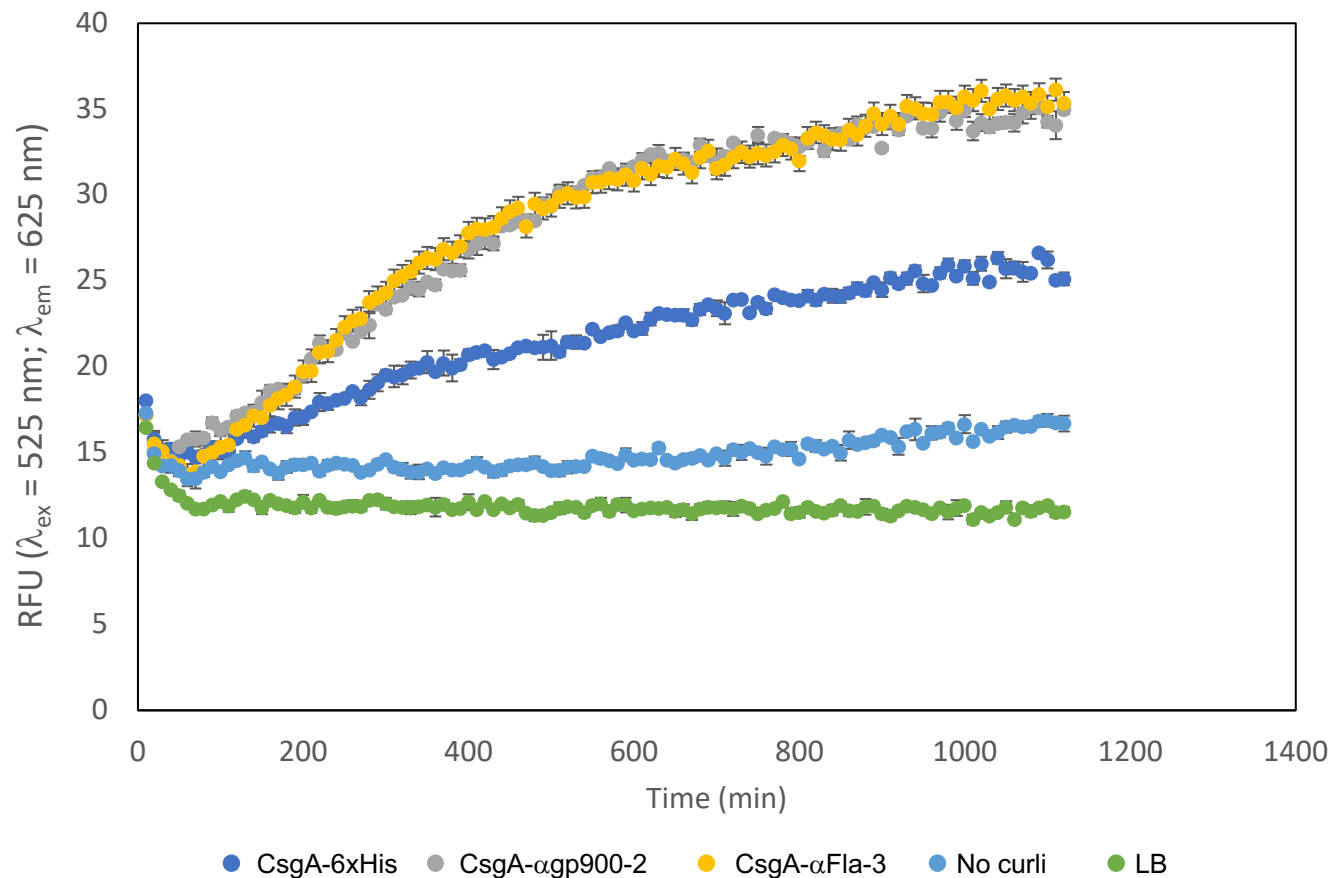

Supplement: S7 Fig — A shift in emission maximum for Congo Red upon binding to curli fibers in growing PBP8 cultures was used to help confirm fiber formation for two CsgA-VHH fusions. “No curli” refers to a negative control wherein PBP8 was transformed with a plasmid bearing the same antibiotic selection markers but no curli genes. “LB” refers to a negative control containing no cells, only LB medium and Congo Red dye. (PDF) [file ppat.1010713.s009.pdf]

**(a)**

CsgA-6xHis

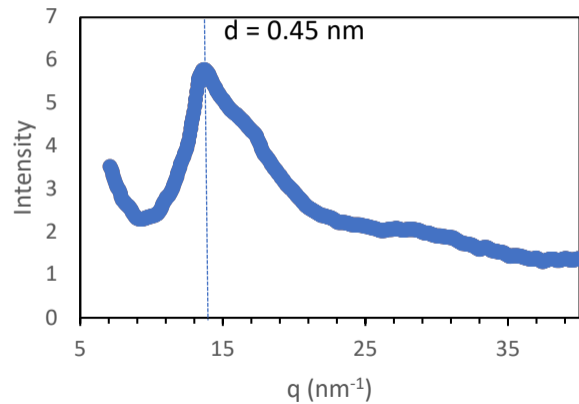**(b)**CsgA- $\alpha$ Fla-3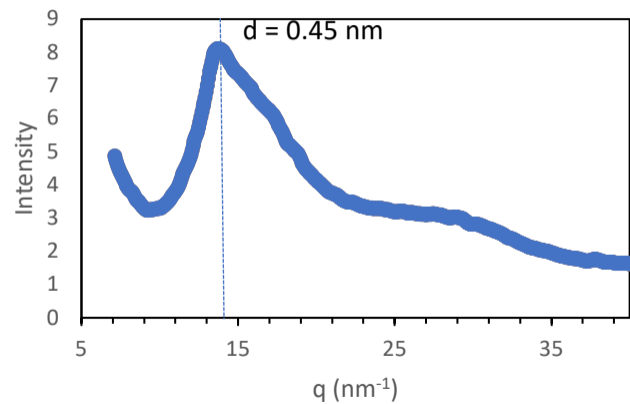**(c)**CsgA- $\alpha$ gfp900-2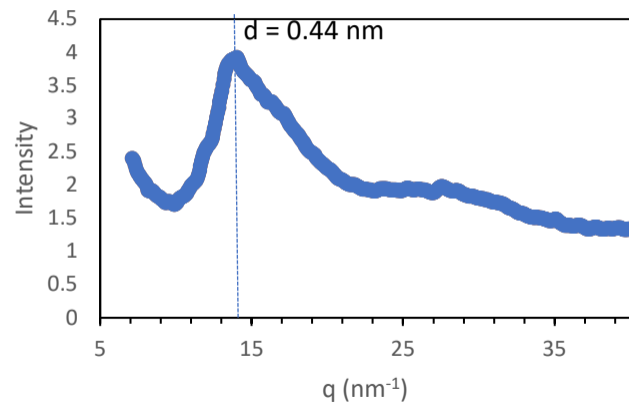

Supplement: S8 Fig — (a) CsgA-6xHis; (b) CsgA-αFla-3; (c) CsgA-αgp900-2. (PDF) [file ppat.1010713.s010.pdf]
