# Supplementary material for: Single domain antibodies against enteric pathogen virulence factors are active as curli fiber fusions on probiotic E. coli Nissle 1917
Source: PLoS Pathog. 2022 Sep 15;18(9):e1010713. doi: 10.1371/journal.ppat.1010713 (PMC9477280; doi:10.1371/journal.ppat.1010713)
Supplement: Attachment — Submitted filename: Rebuttal v6.docx [file ppat.1010713.s011.docx]

Quotes from reviewer critiques are in blue.

**Manuscript Title**: Single domain antibodies against enteric pathogen virulence factors are active as curli fiber fusions on probiotic E. coli Nissle 1917

**Author responses to reviewer critiques**

We thank the reviewers for their time and effort in providing constructive critiques to our work. We note the several positive and encouraging comments were made by each of the reviewers. Below we provide specific point-by-point responses to the reviewer critiques. We provide abbreviated quotes from the reviewer critiques in blue, author responses in black, and quotes of the revised manuscript in italics.

**Reviewer #1**

Critique 1.1: One potential issue is that there does seem to be some effect of the VHH on the morphology of the curli (S3) that may influence functionality. The FESEM suggests differences in structure of the biofilm/curli that may affect the way the VHH is able to function that was not reflected in the congo red binding or ELISA assays. For example, the anti-Cryptosporidium and one of the anti-IpaD did not function as hoped even though those antibodies worked in ELISA and the curli were expressed as shown by congo-red binding. Is there a way to try to evaluate the potential functionality by another assay such as crystal violet staining as is done for biofilm? Would it be reflective, or is the issue more with structures that are attached to the target bacteria such that this approach is best for secreted virulence factors or factors that stick out further from the bacteria such as the flagellin?

CsgA secretion and assembly is extremely robust for a wide range of fusion domains, as shown by the Congo Red binding data in Figure 4E, along with numerous publications from our group and others (references 43 and 44 in the main paper; Duraj-Thatte, et al. *Advanced Materials* 2019, 1901826; Nussbaumer, et al. *ChemCatChem* 2017, 9, 4328). Data in Figures 5-7 in this manuscript also confirm that some VHHs remain functional after fusion to CsgA. Therefore, we posit that neither a lack of secretion nor a lack of assembly explains the examples where the soluble version of a VHH was functional (in terms of bioactivity) but the CsgA fusion was not. Accordingly, biofilm formation assays like crystal violet staining will not reveal any more information than the Congo Red assay. Instead, we think the most likely explanation of these results is misfolding of the VHH domain. Other explanations, like altered curli fiber morphology (e.g. hyper-aggregation), would not seem to abolish all VHH functionality, since some VHH domains would remain exposed in this case. In the case of the anti-IpaD and anti-*Cryptospodirium* VHHs that fit these criteria, we think the hemolysis assay and ELISA, respectively, are the best and most direct measure of VHH functionality, since they can be compared directly to the corresponding soluble VHH experiment. The failure of some VHHs underscores the unpredictability of fusion proteins, which must undergo many biological processing steps in order to perform optimally (transcription, translation, secretion across 2 membranes, extracellular amyloid assembly, VHH folding), and the importance of in vitro screening of CsgA-VHH fusions prior to further work, for example in vivo studies. Furthermore, we do not observe a direct correlation between SEM morphology and function in the data presented here, and a more thorough study of this relationship is outside the scope of this manuscript, in our opinion.

Critique 1.2: There are concerns with Fig 7 & S6. The PBP8 with CsgA-VHH were overlaid onto the sporozoites. Then the fluorescent foci were counted with Image J. If however, the bacteria were clumped together much more for the gp900-1, then the foci would be larger and counted as more--is that correct? There is certainly a lot more red staining for the PBP8gp900-1--is that more binding to the sporozoite, or more bacteria binding to each other? The zoomed in image or are there just more bacteria in those fields. A control of bacteria without the sporozoites might be helpful, as well as a comparison of the FESEM for both of the PBP8 gp900 constructs.

The images shown in Figure S6b show a magnification level that should have enabled the detection of individual bacteria. Therefore, there should not be bacteria present in each field that are not accounted for by the fluorescent foci. Furthermore, the images were analyzed without regard for the size of fluorescent foci, so each focus was counted equally. This scenario underestimates the binding interaction between bacteria and *Cryptosporidium*, since foci that are counted once may represent multiple binding interactions. Finally, it is unclear why there would be bacterial clumping in the case of the gp900-1 fusion but not in the other cases. We contend that the CsgA-anti-GFP construct is the most appropriate control for this experiment. This is supported by additional data we obtained that shows a lack of bacterial clumping in the absence of sporozoites (new Figure S6A).

Critique 1.3: The soluble anti-Int17 was reported to reduce actin accumulation (did not show pedestals). There was a PBP8 construct made with the anti-Int17. Why was the PBP8 CsgA-anti-Int17 not tested in an actin accumulation assay? This is achievable without much additional work and would be a really nice addition to the proof of concept.

We agree that it would have been interesting to screen all of the anti-intimin and anti-Tir VHHs in all of the functional assays. In response to the reviewer’s critique, we conducted experiments to further validate the anti-Int17 construct in soluble form and also as bound to CsgA. We were able to confirm that soluble anti-Int17 was able to block pedestal formation. This new data is included in the revised manuscript as Figure 3j. Unfortunately, technical difficulties, such as an inability to differentiate between EPEC and PBP8 in fluorescence staining experiments, hindered our ability to confirm the same pedestal-blocking function for curli-bound anti-Int17. Given our limited time, personnel, and resources, we are opting to resubmit with the data we have rather than re-engineer the system to enable better characterization. We hope the reviewers are able to see the value in the data we are able to report at this time, which we feel supports the main points of the paper.

Critique 1.4: line 219, S2 and elsewhere--what is shown is actin accumulation, not pedestal formation--though the two are related of course.
We interpret this critique to mean that we did not show the morphological formation of pedestals, for example with electron microscopy. However, the way that we use the term “pedestal formation” is consistent with several literature reports, which routinely use actin staining readouts as a proxy for the formation of pedestal structures.[ [doi.org/10.1128/IAI.01523-13](https://doi.org/10.1128/IAI.01523-13); doi:10.1128/IAI.01523-13[; doi.org/10.3389/fmicb.2011.00226](https://doi.org/10.3389/fmicb.2011.00226)] Therefore, we have opted not to change this nomenclature in the revised manuscript. We did add a clarifying statement (Line 20, revised manuscript):

“*Here, we use actin accumulation from fluorescent staining as a proxy for the formation of pedestal structures, consistent with other publications.”*

Critique 1.5: Figure 4 - most of it unneeded, especially with Figure 1; all of figure 4 could go into the supplemental. Alternatively, Fig 1 could go into the supplemental, since the ultimate goal is the probiotic, not the soluble VHHs.
We received conflicting reviewer comments on the utility of Figures 1 and 4 in the manuscript. We have opted to remove Figure 4A, since it depicts a process occurring at the mucosal gut lining that is not supported directly by our experiments. However, we believe it is important to keep Figure 1 and the rest of Figure 4 in order to make sure that the mechanism of pathogen interference and *E. coli* Nissle engineering is clear to readers from a broad audience.

Critique 1.6: Figure 4 statistical test should be a one-way ANOVA rather than two way--the only variable is congo red binding. Also, Figure 5 -panel A - one-way ANOVA
We thank the reviewer for catching this typographical error. The statistical test performed was actually a one-way ANOVA. The figure caption has been updates to reflect this.

Critique 1.7: line 366 - does the fact that gp900 is shed in trails mean that the antibody would be titered away from the sporozoite? It seems like an antigen that does not shed would be a better target.

We were also concerned with the fact that gp900 is shed. However, it met several other criteria for inclusion in our library. The model of invasion by apicomplexa is through shedding of micronemal proteins (such as gp900) through the parasite surface to establish contact with host cells during locomotion. Specific antibodies to gp900 inhibit *Cryptosporidium* attachment and infection *in vitro* and as such gp900 is considered indispensable for cell invasion (Barnes, 2001 and Cevalos, 2000). Therefore, we argue that gp900 is a good target for EcN in the context of existing mechanistic information about *Cryptosporidium* invasion and its blockage.

- Bonnin A*, et al.* Characterization of a monoclonal antibody reacting with antigen-4 domain of gp900 in *Cryptosporidium parvum* invasive stages. *Parasitol Res* **87**, 589-592 (2001).
- Cevallos AM*, et al.* Mediation of *Cryptosporidium parvum* infection in vitro by mucin-like glycoproteins defined by a neutralizing monoclonal antibody. *Infect Immun* **68**, 5167-5175 (2000).

Critique 1.8: line 28 - delete "scherichia" in front of period so that it reads E. coli
This typo has been fixed.

Critique 1.9: line 58 - "mucus-establishing" means?
The term “mucus-establishing” has been removed.

Critique 1.10: line 139-intimin does vary in the C-terminal third or so--hence whey there are so many different intimin types
This sentence has been amended to say: “Unlike flagella, the EspA, and portions of the intimin, and Tir proteins are relatively well conserved, increasing the likelihood of identifying VHHs that recognize diverse AE pathogens.” This statement is supported by reference 47 in the revised manuscript.

Critique 1.11: line 155 - is "species" meant here, or perhaps "pathotype"? It is confusing because the previous part of the sentence is talking only about *E. coli*. Or is it meant that antibody to *E. coli* intimin does not react with *Citrobacter* intimin?
We agree that the term “species” is confusing here. We have changed the sentence to the one below, which avoids this confusion.

“*Despite our success in identifying several VHHs that recognized purified intimin proteins from the various pathogenic E. coli studied, each VHH was found by ELISA to be highly specific only to intimin from the pathovar on which it was selected (not shown), and, importantly, none recognized E. coli strain MC1061 expressing EPEC or EHEC intimin on the bacterial surface (e.g., αInt-13, Table 1).*”

Critique 1.12: Table 1 footnotes--in "d", "e", and "f" Figure 3 not Fig 2, correct?

We thank the reviewer for catching this. These typos have been fixed.

Critique 1.13: line 190 - bacterial movement diameter?
This term has been amended to read “colony diameter”.

Critique 1.14: line 193 - Table 1 does not give IC50 values. Is Fig 2B meant?
This typo has been fixed. “IC50” has been changed to “EC50”.

Critique 1.15: Figure 4 is referred to out of order in the text. However, see above for other comments about Figure 4.

This has been resolved with the figure restructuring.

Critique 1.16: line 275, 626, supplemental figs S3-5 and elsewhere--EcN is referred to as the strain used, but it really is PBP8--check throughout manuscript.
We have changed “EcN” to “PBP8” when discussing the specific strain used in our experiments. However, we maintain the abbreviation “EcN” in other parts of the paper to indicate relevance to broader Nissle engineering efforts.

Critique 1.17: line 287 Not all S. dysenteriae have Shiga toxin - only serotype 1. Additionally, the antibody shown only neutralizes Stx2, not Shiga toxin.
This sentence has been amended to read: “Shiga toxins are potent bacterial toxins comprising several variants produced by the AE pathogen EHEC, as well as by serotype 1 of *Shigella dysenteriae*“

Critique 1.18: line 350 'tclam"?

This typo has been fixed.

Critique 1.19: line 474 please insert the Table #s

The table numbers have been added.

Critique 1.20: line 544 - add pET32 vector to the supplemental table
This typo has been fixed.

Critique 1.21: line 575 Italicize Escherichia coli here--also check Supplemental Table and citations for italics.
These typos have been fixed.

Critique 1.22: Figure S3-it would be nice to have a figure of PBP8 expressing CsgA with no VHH.

We have previously published the results requested by this critique (Praveschotinunt, Nature Communications 2019, 10(1), 5580 – Figure 2D). We have amended the main text to reflect this. At the end of the section entitled “Design and expression of curli-VHH…”:

“All CsgA-VHH constructs tested resulted in the formation of curli material with morphology similar to the wild-type curli fibers, as produced and assembled by PBP8 based on previously published results (Fig 4d, S3 Fig).[REF 43]”

16. Why is simulated colonic medium used for the GFP pull down study?

The decision to perform this pull-down experiment in simulated colonic fluid was made at an early stage of the project, when we thought it would be useful for in vivo translation. However, we do not think it is necessary to amend or augment this piece of data in retrospect, given the multiple published instances from our lab (Birnbaum, et al. *Advanced Science* 2021, 2004699) and others (van Gerven, et al. Molecular Microbiology 2014, 95(5), 1022) of CsgA-fused VHHs pulling down their target (including GFP) under a wide range of buffer conditions. Therefore, we expect this GFP pulldown to also work under a wide range of conditions.

End review

**Reviewer #2**

Critique 2.1: The paragraph spanning lines 94-101 describes potential issues for the application of the present work. It should either be moved to the Discussion section or omitted entirely.
We think the paragraph is necessary in its original context to provide context for the weaknesses of direct administration of VHHs to gastrointestinal tract and justify the need for engineered living systems as a delivery mechanism.

Critique 2.2: The phrase “with an IC50 as low as 1.0 μM (Table 1)” found on line 192-193 calls out to Table 1 where EC50’s are displayed. It is also a little confusing which EC50’s are being referred to.
This typo has been fixed.

· The section title “Specific anti-Tir and anti-intimin VHHs block the interaction between intimin and Tir” suggests that results associated with anti-intimin VHHs would be discussed. However, Fig 3a only shows data including anti-Tir VHHs. This data should be included.
The section title has been updated to read: “Specific anti-Tir VHHs block the interaction between intimin and Tir” We do not have data showing anti-intimin VHHs in this context.

· The sentence “This clustering promotes F-actin assembly beneath bacteria bound at the host plasma membrane and results in the formation of actin pedestals that facilitate pathogen colonization” on line 212-214 should have a reference.
This reference has been provided in the revised manuscript (reference 9).

· The VHH called αTir8 was shown to be very effective at blocking the Tir-intimin interaction in vitro (Fig 3a). Why was αTir8 not used in the in vivo fluorescence assays shown in Fig 3b? Similarly, why does αTir13 perform well in vivo and yet does not block the interaction in vitro?
This is a valid point based on the data presented. We wish we had the resources to make and screen all the VHHs we developed. In actuality, the data represents the efforts of several generations of researchers across multiple research groups and institutions. As such, it was not possible to screen every candidate and we collected the best data we had at the time and present it here. We think the data that we present still supports the foundational conclusions that our VHHs can block pathogen-host interactions in ways that could be useful therapeutically.

· Fig 4a was never referred to.
Figure 4a was removed.

· The paragraph on lines 353-361 is not results and should be moved to the Introduction or Discussion section.
We concede that the paragraph in question technically does not report results. However, we have opted to keep it in its original location in the amended draft for the sake of readability. We contend that moving this paragraph to the introduction or discussion would require even more text to remind the reader of the Cryptosporidium context.

· Fig S6a and S6b seem to be redundant, only S6b is necessary.
While these two figures indeed represent the same conditions under different magnification, they support different claims. Fig. S6a supports quantitative analysis for Fig. 7c. We have added text to the caption to clarify this. Fig. S6b show co-localization of the signals from EcN and sporozoites.

· It would be beneficial to reader comprehension if some figure references would be included in the Discussion section.

We have added Figure callouts to the Discussion section where relevant.

**Reviewer #3**

Critique 3.1: Although the study is exciting and has potential for in vivo delivery of inhibitory VHHs to block virulence in several bacterial species, critical data is missing to confirm that this delivery platform could be used in vivo. It would be powerful, instead of showing that the system is working for blocking several pathogens, to show that for the three initial virulence factor that this system could be used in vivo.

Indeed, we are excited to demonstrate the in vivo efficacy of this system. However, considering the amount of data already in this manuscript (this reviewer refers to is as “dense” already) and the time, funds, and interdisciplinary coordination required to run a proper set of experiments to confirm in vivo functionality, we are opting not to include in vivo data in this paper and follow up with in vivo data in a subsequent submission.

Critique 3.2: Furthermore, no mechanistic information is included on how CsgA-VHH fibers are assembled. It would be informative to show whether the VHH fibers hinder with the amyloid properties of curli or if curli can still form the amyloid fibrils. As the paper is dense, it is hard to follow the manuscript and it would help if more details on methods, strains and growth conditions are provided.

We agree that the fundamental characterization of modified curli fibers in this manuscript is abbreviated. However, this was a conscious decision on our part not to replicate curli engineering data that has been presented in our previous publications. We and others have created numerous fusions and mutations to CsgA without abrogating its ability to be produced recombinantly, secreted, and assembled into amyloid fibers. We have demonstrated, for example, that many CsgA fusions maintain their amyloidogenic signatures (e.g. fluorescent birefringence – Nguyen, et al. Nature Communications 2014, DOI:10.1038/natcomms5945; WAXS – Duraj-Thatte, et al. Nature Chemical Biology 2021, DOI:10.1038/s41589-021-00773-y). Figure S3a shows that, in the absence of CsgA induction there is no fiber formation from PBP8. This ability is also supported by the data in Figures 4 and 5 of this manuscript. We think that the Congo Red data, combined with the fact that there are clearly fibers present in the SEM images of all the other constructs is sufficient evidence of fiber formation for this manuscript. While we do think it is likely that these fibers are amyloids based on our previous work, their amyloid nature it is not essential to the main point of this manuscript.

Critique 3.3: As the paper is dense, it is hard to follow the manuscript and it would help if more details on methods, strains and growth conditions are provided.

We are unsure of how to address this critique most directly. With regards to methods, we sought to provide sufficient detail in the Methods section to reproduce experiments, including growth conditions. A complete list of strains is present in the amended Table 1. If the reviewer has additional specific requests, we would be happy to address them.

Critique 3.4: 1-Authors speculate the curli would be expressed in the intestinal tract but there are no studies that showed that E. coli Nissle expresses curli. Again Fig4A is speculative and should be removed. It would strengthen the manuscript to investigate at least for one of the virulence factors that the curli-VHH display system would work in vivo rather than showing it works for all in vitro.
We reiterate our interest in demonstration in vivo for this system. However, we maintain that it is outside the scope of this manuscript, as explained above (Critique 3.1). We think our track record of publications showing the use of very similar engineered microbial systems (i.e. composed of E. coli Nissle-derived PBP8) is sufficient to convey the feasibility of downstream in vivo implementation. For example, we already have multiple published confirmations that PBP8 can be used to produce engineered curli fibers in the mouse GI tract (Kan, et al. ACS Synth. Biol. 2021, 10, 94−106; Praveschotinunt, et al. Nature Communications, DOI:10.1038/s41467-019-13336-6).

Critique 3.5: Alternatively, more information on the structural assembly or the interactions of the curli-VHH display system with virulence factors could be presented. Although SEM pictures are showing the expression of curli, it is important to provide data on whether curli can still assemble into amyloid fibrils while carrying VHH. Thioflavin T based amyloid assay supported by TEM or Circular dichoism would be powerful to show whether curli can still assemble into a fibrillar conformation. Are there any data to support the VHHs are displayed as depicted in Fig4C? This figure needs to be supported by additional data to show the mechanism of action.

As explained above, we feel that the Congo Red data combined with the SEM images (both with appropriate negative control experiments) are sufficient to demonstrate extracellular fibrilization for our system. While we do think the fibers are likely to be amyloids, their amyloidogenic nature is not essential to this manuscript and has been characterized extensively for similar fusions in other publications. We have shown in several previous publications that a wide range of fusions (including those of comparable size to VHHs) still allow for amyloid formation (Duraj-Thatte, et al. *Nature Chemical Biology* 2021 DOI: 10.1038/s41589-021-00773-y).

Critique 3.6: Regarding Table 1. This is a nice summary of the results on a big data set. However, it is important also for the reader to understand how this data is generated with appropriate example. For instance show data on how the EC50 values were determined. Not all data but selective VHHs can be showed as graphs to support the tables. The rest can be left in the supplementary data

We agree that it is important that we more clearly explain how the EC50 values were determined, and we have thus added a paragraph to the Methods section entitled Dilution ELISAs. We also refer readers to this section in the legend to Table 1.

Critique 3.7: Fig1 is not very useful as the authors investigate virulence factors of several different pathogenic bacteria in the manuscript. This figure could be moved to a model slide at the end if needed.
We opted to keep Figure 1 as is in the modified manuscript. Even though it only depicts a subset of pathogenic interactions compared to all of those addressed in the paper, we feel it will help to facilitate conceptual understanding of relevant mechanisms among a broad audience.

What is the reasoning in using curli as a display system for VHHs? Can authors add this information in Ln244.
We have added text to clarify the advantages of curli display at the location suggested by the reviewer. To summarize, curli display offers: 1) multivalency, which could enhance the ability to block pathogen-host interactions; 2) high local concentration/enhanced residence time near the site of pathogen colonization; 3) the possibility to physically entrap pathogens within a nanofibrous mesh; and, 4) the ability to replenish the VHH supply to compensate for proteolytic degradation and gut clearance by flow. Some of these features already appeared in the original discussion. We have added text to the revised manuscript to highlight the other features of curli display – line 435 (revised manuscript):

“This *in situ* production could also have the benefit of a high local concentration near the site of pathogen colonization.”

Are there any supporting data to show that the VHHs are displayed as depicted in 4C?
Figure 4C is an idealized schematic that is representative of current theories about curli secretion and assembly that can be found in the literature. In the SEM images provided in the manuscript (Figures 4D, S3), the fibrous nature and approximate thickness of the fibers can be clearly observed. Since the fibers are composed solely of CsgA-VHH fusions (or CsgA only), we think the schematic in Figure 4C is a reasonable representation, even though certain specifics (e.g. all the fusion domains on one side of the fiber) are not currently supported by any structural data. Furthermore, we have previously published many papers that describe a similar fibrous display behavior with a multitude of CsgA fusions, including several that explicitly present data that supports their amyloid nature (Nguyen, et al. *Nature Communications* 2014, 5(1), 4945; Duraj-Thatte, et al. *Nature Chemical Biology* 2021 DOI: 10.1038/s41589-021-00773-y). We have amended the text at the end of the section entitled “Design and expression of curli-VHH…”:

“Previous work has confirmed the tolerance of the curli system to a wide range of CsgA fusion proteins without negatively affecting amyloid fiber assembly.”

Furthermore, Figure 4C is in line with other depictions of curli fiber assembly in the literature (Barnhardt, et al. Annual Review of Microbiology, 2006).

Fig 4A is not referred in the text. This figure is not needed where it is. Also the authors do not show any data suggesting that curli would be expressed through the epithelial lining and trap bacteria. This is speculative. Maybe move 4C to 4A and make 4B bigger so that the cloning strategy is visible.
This has been resolved with the figure restructuring.

4E no IPTG control is missing. CR also binds to cellulose. It is important to add the controls to the assay.
The “No CsgA” control experiment should address the need to account for cellulose binding, as it is the same strain as the others, but harbors a plasmid that contains no gene encoding for CsgA. Cellulose production should be the same in this condition versus the rest of the conditions. We have added the text below to the relevant section in the revised manuscript.

“*The “No CsgA” negative control condition also confirms that background signal arising from non-specific Congo Red binding (for example, to cellulose) is accounted for and does not affect the overall conclusions.*”

4D. show also no IPTG control (or CsgA) in the SEM picture to show that there is no CsgA expression.
The image in Figure S3A shows that in the absence of CsgA expression (i.e. bacteria harboring a plasmid without a gene encoding for CsgA), there are no fibers.

Ln 425. Add reference to the statement that ECN expresses curli in the intestinal tract. Has this been shown?
We have previously demonstrated and reported on the ability of engineered EcN to produce curli fibers in the GI tract of mice. This reference has been added to support the statement. Notably, our engineered EcN strains produce curli fibers in response to either temperature-based or chemical induction, rendering curli production independent from any native processes related to biofilm formation.

We have amended the Discussion section to reference the relevant publications from our group.

Add details for panning into the material methods for the generation of VHHs.

More details on the panning procedures have been added to the Methods section with some specifics added to Table 1
