# Supplementary material for: Single domain antibodies against enteric pathogen virulence factors are active as curli fiber fusions on probiotic E. coli Nissle 1917
Source: PLoS Pathog. 2022 Sep 15;18(9):e1010713. doi: 10.1371/journal.ppat.1010713 (PMC9477280; doi:10.1371/journal.ppat.1010713)
Supplement: Attachment — Submitted filename: Rebuttal Revision2 v3.docx [file ppat.1010713.s012.docx]

Quotes from reviewer/editor critiques are in blue.

Our responses are in black. We thank the reviewers and editor for their time.

From Editor:

One outstanding question is whether the modified curli are expressed in the gut or settings that resemble the gut environment. At the very least, the limitations of your study need to be appropriate discussed, which is lacking in the current manuscript. When developing these revisions, please play close attention to the remarks of Reviewer 3. In cases where experimental work was proposed or requested, your response letter should clearly describe either (i) why you feel the work is outside the scope of this manuscript and/or (ii) the technical limitation that would prevent the experiment from being done.

It is true that we do not include data in this manuscript that demonstrates that curli fibers are produced in vivo in the gut environment. However, this was a conscious decision made for several reasons. First, we have previous published data demonstrating that curli fibers can be produced in the mouse gut using the exact *E. coli* Nissle-derived chassis used in this paper (PBP8). These demonstrations monitored curli fiber production in homogenized fecal pellets over time with single or repeated oral administrations to mice, with various antibiotic treatment protocols (concurrent antibiotics to which our strains were resistant, and antibiotic pre-treatment only), with different gene induction systems (chemical inducers co-administered in drinking water and temperature-based induction), and with different CsgA fusion proteins (including fusions to VHH domains). These publications also contain indirect evidence supports the fact that curli fiber production in vivo, including ways in which different curli fiber constructs, produced from the same chassis, influence host biology, trafficking of fibers in the GI tract, and bacterial residence times in vivo. We think this is ample evidence, obtained across multiple years with different researchers and in different publications, that it is feasible to engineer *E. coli* strains to produce curli fibers in vivo. We do not think it is necessary to repeat such demonstrations for the specific CsgA-VHH fusions discussed in this paper. With the resources we had to pursue the aims of this project, we tried some pilot experiments in which we co-administered EPEC and engineered *E. coli* Nissle. However, interpretation of this data was more challenging than anticipated, so we opted not to include it in the manuscript. We recognize the need to demonstrate efficacy of our system in protecting against infection in vivo. However, since it will require further bacterial engineering, we will have to seek more funding to pursue this work with the resources it necessitates.

We concede that there could be unpredictable outcomes with the specific CsgA-VHH fusions we include in this manuscript. We have accordingly added the following text to the discussion of the revised manuscript:

“*This work would benefit from further structural characterization of CsgA-VHH fibers to understand their morphology and confirm their functional production in vivo.*”

Reviewer #1: Overall the authors addressed the concerns raised.

We thank the reviewer for their time in reviewing our manuscript.

Reviewer #3: Manuscript by Gelfat et al investigates the engineered VHHs, the variable domains of camelid heavy-chain-only antibodies as ways to inhibit the virulence of multiple pathogenic enteric bacteria. Although this is an exciting study and authors did not address the comments raised by the reviewers. Many comments were left hanging without showing any further data. Authors argue that the some of the information could be found in their previous work. However, the previous studies does not exactly addressing what the reviewers were asking for. Furthermore there are many speculations raised by the authors. Although the reviewers pointed out these concerns were not sufficiently addressed and not discussed as weaknesses of the study or as future directions in the discussion section.

We thank the reviewer for their help in improving this manuscript. We respectfully disagree on the specific point regarding engineered curli fiber production in vivo. A detailed response is provided above to a similar critique from the Editor. We agree that we should be clear about the potential weaknesses of our approach, as raised by the reviewers. Accordingly, we have added text to the revised manuscript that discusses these weaknesses.

**Part II – Major Issues: Key Experiments Required for Acceptance**
Please use this section to detail the key new experiments or modifications of existing experiments that should be absolutely required to validate study conclusions.

Generally, there should be no more than 3 such required experiments or major modifications for a "Major Revision" recommendation. If more than 3 experiments are necessary to validate the study conclusions, then you are encouraged to recommend "Reject".

Reviewer #1: (No Response)

Reviewer #3: -There are no structural data shown in the manuscript that the CSG-VHH fibers are assembled into functional entities. Congo red assay that is not specific and one SEM picture is not sufficient to argue that these engineered constructs are working in the way that is proposed in the manuscript. Although SEM pictures are showing the expression of curli, this method is not specific enough to show structural changes. More thorough study of this relationship is not outside the scope of this manuscript. Instead it is needed to confirm that the engineered VHHs are working in the way that they are proposed by the authors. It was suggested in the previous revision to utilize Thioflavin T based amyloid assay supported by TEM or Circular dichroism to confirm the claims done by the authors.

Our original proposition was that the combination of Congo Red assays and SEM showing fiber morphology was sufficient structural characterization for this manuscript. We don’t think our schematic depiction of curli fiber assembly in Figure 4 is overly prescriptive. It is in accordance with several other publications regarding curli fiber characterization. Nor does the success of our approach depend significantly on maintaining an amyloid-like structure for the secreted CsgA-VHH fusions. The combination of the SEM images (Figure 4C) and the Congo Red data (Figure 4D) should be sufficient to claim that our secreted CsgA-VHH fusions self-assemble into fibers. However, in an attempt to increase the rigor of our assertions with respect to this critique, we have performed further experiments to confirm the structural characteristics of a sub-set of CsgA-VHH fusions reported in this paper.

First, we have performed a kinetic assay demonstrating curli amyloid expression in growing cultures using Congo Red fluorescence as a readout. We think this is a sufficiently close approximation to the reviewer’s request for ThT assays. We opted not to perform ThT assays because of the considerable difficulty of isolating pure, unassembled CsgA and CsgA fusion proteins, which requires disassembly under very harsh conditions (1:1 TFA/HFIP) followed by affinity purification under denaturing conditions and dialysis immediately prior to re-assembly. After all this, there is no guarantee that the CsgA-VHH fusion will behave in the same way that it does when produced by the cell. We stress that the in situ Congo Red fluorescence assay we report here should not be considered equivalent to the endpoint Congo Red absorbance measurements reported in Figure 4D. We have previously demonstrated the viability of using a shift in Congo Red emission spectrum to specifically monitor curli fiber production in growing cultures [Kan, et al. Applied and Environmental Microbiology 2019 <https://doi.org/10.1128/AEM.00434-19>]. Notably, we specifically validated this method using ThT signal as a comparison. Although ThT excelled for the characterization of CsgA assembly from purified monomers, it was less effective than Congo Red for monitoring curli fiber production in growing bacterial cultures. The technique was also validated with multiple *E. coli* strains, including those derived from *E. coli* Nissle. Our results with two CsgA-VHH fusions reported in this manuscript (CsgA-αgp900-2 and CsgA-αFla-3) demonstrate that they are produced under inducing conditions by strain PBP8 with comparable kinetics and fluorescence intensity as a wild-type CsgA sequence. A negative control consisting of PBP8 bearing a plasmid containing the same antibiotic resistance gene but no curli genes shows minimal background signal during this experiment. These data are now included as Figure S7 in the revised manuscript and the main text has been revised to discuss the results.

Second, we have performed wide-angle X-ray scattering (WAXS) studies on dried films composed of the same two CsgA-VHH fusion constructs. We have previously reported that similar dried films exhibit two characteristic peaks at 0.46 nm and 0.98 nm, which is indicative of the interstrand spacing and intersheet spacing of amyloids, respectively. [References 1) <https://doi.org/10.1038/s41589-021-00773-y>, 2) DOI: 10.1039/c6cs00542j, 3) http://dx.doi.org/10.1098/rsfs.2016.0160]. The films composed of CsgA-αgp900-2 and CsgA-αFla-3 exhibited the same peak at 0.44-0.45 nm, suggesting that they too are amyloids. These data are now included as Figure S8 in the revised manuscript and the main text has been revised to discuss the results. Furthermore, the fact that we were able to form films at all using a previously reported filtration-based protocol for curli fiber isolation suggests that the fusion proteins are able to fibrillate. Cells that are not producing curli fibers do not form films on the filter surface using our protocol because they have no fibrous network to prevent passage of the cells and soluble proteins through the filter pores.

We think that these two additional characterization techniques should be sufficient supplementary evidence to confirm the self-assembly and amyloid nature of our fibers that mirrors the behavior of curli fibers composed of unmodified CsgA.

-Authors speculate the curli would be expressed in the intestinal tract but there are no studies that showed that E. coli Nissle expresses curli. The publications that were brought up by the author (Kan, et al. ACS Synth. Biol. 2021, 10, 94−106; Praveschotinunt, et al. Nature Communications, DOI:10.1038/s41467- 019-13336-6) does not directly show that curli is expressed by E. coli Nissle in vivo. The methods used in these previous studies are indirect methods showing E. coli colonization.
We refer to our earlier response to this critique that cites several other examples from our won work that confirm curli fiber production (from various CsgA fusion proteins) inside the mouse GI tract.

-It is necessary to show that at least for one of the virulence factors that the curli-VHH display system would work in vivo to confirm that the system is functional.
We are also eager to demonstrate the efficacy of our system with an in vivo infection model. However, we do not currently have the resources to do so. We think that the body of data presented in this manuscript is still strong enough to suggest the promise of our platform for further development – a proposition that seems to be supported by the other two reviewers.

-Although Figure S3a shows that, in the absence of CsgA induction there is no fiber formation from PBP8, this doesn’t prove that the VHHs constructs are assembled into right conformation. It only suggest that they are exported and assembled in some fashion. Previous studies shown that CsgA fiber diameters can change in case of wrong assembly. Is there any data to support that the CsgA fiber width is the same in the presence of VHHs? Instead of referring to approximate thickness, measurements can be made as suggested by the reviewers using TEM in the previous submission. Additionally, previously published CsgA fusions were different that the VHH constructs that are being studied in this manuscript. Therefore, it is not relevant to cite publications that used very different structural conformations instead of showing data that would help this manuscript.

We accept the reviewer’s point that Figure S3 does not conclusively prove that the CsgA-VHH fusions are assembled into amyloids in a manner that is analogous to wild-type curli fibers. In our revised manuscript, we have supplied additional data that supports the amyloid-like nature of our fusion proteins, in accordance with previous reports with several other CsgA fusion proteins. We have also adjusted the language used in the manuscript to avoid specific reference to amyloids and use the term “fibers” instead. However, we disagree that this point is important to the overall functional performance of our platform. The key point that we are trying to convey is that our engineered bacteria is able to create protein fibers that display functional VHH domains. This point is supported by several data presented in the manuscript. The fibrous nature of our CsgA-VHH fusion proteins is supported by Figures 4cd, S3, S4, and the new Figures S7 and S8. The functionality of the CsgA-VHH fusions is supported by Figures 5, 6, 7, S4, S5, and S6. The validity of these results has been recognized by the other reviewers and this reviewer has not raised critiques about the functionality of the CsgA-VHH fibers. We think the claims in our revised manuscript are therefore well supported by the data that we present.

**Part III – Minor Issues: Editorial and Data Presentation Modifications**
Please use this section for editorial suggestions as well as relatively minor modifications of existing data that would enhance clarity.

Reviewer #3: Authors argue that Figure 4C is an idealized schematic that is representative of current theories about curli secretion and assembly that can be found in the literature. Please refer Figure 4C as an idealized schematic as the data is not provided in this manuscript to support that he VHH constructs are assembled in a similar fashion to native CsgA.

We have amended the language in the caption and the main text to emphasize the idealized nature of the schematic in Figure 4C.
